# Efficient Solar Desalination of Seawater Using a Novel Carbon Nanotube-Based Composite Aerogel

**DOI:** 10.3390/ma16175815

**Published:** 2023-08-24

**Authors:** Shuai Liu, Shun Wang, Shunxu Shuai, Yuyan Weng, Fengang Zheng

**Affiliations:** 1 Jiangsu Key Laboratory of Thin Films, School of Physical Science and Technology, Soochow University, Suzhou 215006, China; 20204208039@stu.suda.edu.cn (S.L.); wengyuyan@suda.edu.cn (Y.W.); 2 SJTU-Pinghu Institute of Intelligent Optoelectronics, Jiaxing 314200, China

**Keywords:** CNT, carboxymethyl cellulose, photothermal materials, solar steam generation, aerogel

## Abstract

Solar desalination of seawater is an effective approach to address the scarcity of freshwater resources. For solar steam generation, it is critical to design biodegradable, sustainable, low-cost, and high-evaporation-rate technology. This study aims to develop a novel solar desalination technology by designing and fabricating a nanocomposite material with excellent light absorption and thermal conversion properties. We designed a double-layer aerogel structure, which uses naturally abundant carboxymethyl cellulose (CMC) as the basic skeleton to achieve sustainability and biodegradability, and uses carbon nanotubes as the photothermal material for efficient light absorption to prepare a ferric tannate/carbon nanotube/carboxymethyl cellulose composite aerogel (FT-CNT-CMC aerogel). Experimental results demonstrate that the FT-CNT-CMC aerogel exhibits a high light absorption rate of 96–98% within the spectral range of 250–2400 nm, showcasing remarkable photothermal conversion performance. Under a sun intensity of 1 kW·m^−2^, the FT-CNT-CMC aerogel achieves a significant evaporation rate of 1.942 kg·m^−2^·h^−1^ at room temperature. Moreover, the excellent performance of the FT-CNT-CMC aerogel is validated in practical seawater desalination and organic dye wastewater purification. The FT-CNT-CMC aerogel exhibits a retention rate exceeding 99% for Na^+^, Mg^2+^, K^+^, and Ca^2+^ ions in simulated seawater, while no characteristic absorption peaks are observed in methylene blue and rhodamine B dye solutions after purification. These findings highlight the promising potential of the FT-CNT-CMC aerogel in the field of novel solar desalination, providing a viable solution to obtain freshwater.

## 1. Introduction

Freshwater resources play a crucial role in the advancement of human society. As the population, economy, industrialization, and water pollution intensify, the demand for freshwater resources continually increases. Consequently, the scarcity of freshwater has emerged as one of the most pressing global challenges. Seawater desalination and sewage purification through solar steam systems have been proven to be effective approaches in addressing the shortage of clean water [1,2,3]. To achieve high-efficiency solar steam generation (SSG), two critical factors need to be considered: enhancing solar thermal conversion capacity and minimizing heat loss [4]. Substantial advancements have been made in both areas, and some absorbers with various well-designed configurations have exhibited efficient solar energy absorption [5,6].

To enhance the solar thermal conversion capability, various materials, including plasmonic metal nanoparticles [7,8], semiconductor materials [9,10,11,12], and carbon-based materials [13,14,15,16,17,18,19,20], have been utilized as light absorbers. Plasmonic metal nanoparticles possess strong light-to-heat conversion abilities, making them suitable for solar energy collection. However, their narrow-band absorption characteristics often result in a limited light absorption capacity. Enhancing their light absorption across a broader wavelength range through various strategies can lead to increased costs. In contrast, low-cost semiconductor photothermal materials exhibit excellent light-absorbing properties and have been widely employed in solar evaporation systems [19].

In recent years, a new class of two-dimensional material called MXene has shown promise as a light absorber in solar evaporation systems. MXene demonstrates exceptional hydrophilicity and high-efficiency absorption properties across the entire sunlight spectrum. Utilizing two-dimensional MXene flakes as light absorbers has obtained an evaporation rate of 1.31 kg·m^−2^·h^−1^ at 1 sun irradiation [20]. However, the reflection and scattering of incident light on the surface of these flakes hinder the light absorption. The performance has been enhanced by employing MXene aerogel, which has achieved an evaporation rate of 1.46 kg·m^−2^·h^−1^ [21]. This improvement is attributed to the improvement of light absorption within the aerogel pores. Nevertheless, the synthesis of MXene using highly toxic chemicals, such as hydrofluoric acid, hampers its widespread adoption as a light absorber. In comparison to plasmonic metal nanoparticles and semiconductor photothermal materials, carbon-based materials, including polymers [15], graphene [18], and carbon nanotubes (CNTs) [22], have a considerable abundance and cost-effectiveness. Extensive research has been conducted on solar evaporation systems employing various carbon-based materials as light absorbers. Among them, polypyrrole-based solar evaporation systems achieve a water evaporation rate of 1.59 kg·m^−2^·h^−1^ under 1 sun irradiation [23]. Similarly, carbon nanotubes used as light absorbers yield a water evaporation rate of 1.58 kg·m^−2^·h^−1^ under 1 sun irradiation [24]. Ideally, photothermal materials should possess excellent thermal conductivity and light absorption properties across the entire solar spectrum, while also being cost-effective and environmentally friendly. Carbon nanotubes are recognized as a promising photothermal material.

To minimize heat loss during the SSG process, solar evaporation at the air–water interface has been proposed and proved in the past few years [25]. However, this method also creates direct contact between the solar steam power generation system and a large amount of water, leading to significant heat dissipation [26,27]. While some methods have been proposed to reduce heat loss by reducing the contact area between the system and a large amount of water, the heat loss from the substantial water volume may still significantly restrict the further improvement of the evaporation performance [28,29]. To address this issue, we employ a solar evaporation system that entirely isolates a large amount of water from the evaporation structure, thereby minimizing heat loss. 

In this study, we designed a double-layer aerogel structure, taking the natural rich carboxymethyl cellulose (CMC) as the basic skeleton to achieve sustainability and biodegradability, and used carbon nanotubes as the photothermal material to prepare a ferric tannate/carbon nanotube/carboxymethyl cellulose composite aerogel (FT-CNT-CMC aerogel). The developed aerogel exhibits exceptional light absorption properties, with an absorption rate exceeding 96% within the 250–2400 nm range. Furthermore, the evaporation rate achieved reaches an impressive 1.942 kg·m^−2^·h^−1^ under 1 sun irradiation. Notably, the composite aerogel possesses several advantageous features, including a low cost, renewable and environmentally friendly materials, and a simple preparation process. These characteristics make it highly promising for applications in solar desalination of seawater and sewage treatment.

## 2. Experimental Section

### 2.1. Materials

FeCl_3_·6H_2_O (AR), tannic acid, sodium carboxymethyl cellulose, D-(+)-gluconic acid-lactone (GDL), ammonia solution (25%), methylene blue (MB), rhodamine B (RHB), stearic acid, and carbon nanotubes were purchased from Aladdin Reagents Co., Los Angeles, CA, USA.

### 2.2. Synthesis of the CNT-CMC Aerogel

**Figure 1:**
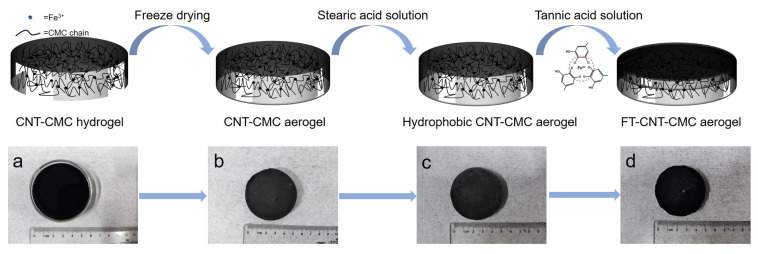
Upper: preparation process of FT-CNT-CMC aerogel. Lower: sample photographs corresponding to the flowchart: (**a**) CNT-CMC hydrogel, (**b**) CNT-CMC aerogel, (**c**) hydrophobic CNT-CMC aerogel, and (**d**) FT-CNT-CMC aerogel.

In the first step, 0.441 g of gluconolactone and 0.75 g of sodium carboxymethylcellulose were added to 25 mL of deionized water and stirred for 3 h. In the second step, a specific quantity of carbon nanotubes was dispersed in 50 mL of deionized water and stirred to achieve concentrations of 5 mg/mL, 10 mg/mL, 15 mg/mL, 20 mg/mL, and 25 mg/mL. Next, 0.435 g of FeCl_3_·6H_2_O was added, and the solution’s pH was adjusted to 7 using ammonia water. In the third step, the two solutions were mixed and stirred for 5 min. The resulting mixture was poured into a Petri dish and allowed to gel for 1 day to obtain CNT-CMC hydrogel, as shown in Figure 1a. Afterward, the gel was placed in a refrigerator and frozen for 12 h. In the fourth step, the carbon nanotube/carboxymethyl cellulose hydrogel was subjected to vacuum freeze-drying at −70 °C for 40 h, yielding the carbon nanotube/carboxymethyl cellulose aerogel (CNT-CMC aerogel), as shown in Figure 1b.

### 2.3. Synthesis of the FT-CNT-CMC Aerogel

In the first step, 0.75 g of stearic acid was added to 50 mL of ethanol and sonicated for 20 min to ensure complete dissolution. Subsequently, the CNT-CMC aerogel prepared earlier at different concentrations was immersed in the stearic acid solution for 12 h. The soaked CNT-CMC aerogel was then dried in an oven at 80 °C for 20 min to obtain a hydrophobic CNT-CMC aerogel, as shown in Figure 1c. In the second step, the upper surface of the hydrophobic CNT-CMC aerogel was immersed in a 2 mg/mL iron tannate solution. This resulted in the formation of an upper layer consisting of ferric tannate/carbon nanotube aerogel (referred to as FT-CNT aerogel), while the bottom layer remained as CNT-CMC aerogel. Thus, the FT-CNT-CMC aerogel was successfully prepared, as shown in Figure 1d.

### 2.4. Characterization

The microscopic morphology and elemental composition were analyzed using scanning electron microscopy (SEM, Hitachi S4800, Tokyo, Japan) coupled with energy-dispersive X-ray spectroscopy (EDS). The thermal stability of the materials was assessed using a thermogravimetric analyzer (TGA, DTA7300, Tokyo, Japan). Reflectance measurements were performed using an ultraviolet-visible-near-infrared (UV-Vis-NIR) spectrometer (UV-3600, Shimadzu, Kyoto, Japan) equipped with an integrating sphere. The absorbance of opaque objects was calculated using the formula A = 1 − R, where R represents the reflectance. Contact angles (WCA) were determined, and videos of the measurements were captured using an optical contact angle meter (DSA255, Kruss, Hamburg, Germany). Absorption spectra were recorded using an ultraviolet-visible transmission spectrometer (UV2550, Shimadzu, Japan). The concentrations of salt ions in simulated seawater and collected water were measured using inductively coupled plasma emission spectrometry (ICP, Optima 8000, Perkin Elmer, Walthman, MA, USA). Infrared images were obtained using a thermal imager (Fluke, PTi120, Everett, WA, USA).

### 2.5. Solar Evaporation Measurement

A solar simulator consisting of a xenon lamp (CEL-S500, AM1.5) and an optical power meter (CEL-NP2000) was employed in this study. A 1.5 cm × 1.5 cm square sample was subjected to 1 sun irradiation for water evaporation. To supply water to the evaporation layer of the sample, a syringe was used for drip irrigation from above, as illustrated in Figure S1. The mass change of water during evaporation was measured using a precision balance with an accuracy of 0.001 g.

## 3. Results and Discussion

With increasing concentrations of carbon nanotubes, the initially prepared CNT-CMC aerogel gradually exhibited a black coloration (Figure S2a–e). Notably, during the increase in CNT concentration from 5 mg/mL to 20 mg/mL, the blackening effect on the CNT-CMC aerogel became more pronounced compared to the previous concentrations. Scanning electron microscopy coupled with EDS was employed to analyze the CNT-CMC aerogel under different CNT concentrations (Figure S2f). The results showed that as the CNT concentration increased, the proportion of carbon atoms within the CNT-CMC aerogels also increased. Specifically, the carbon atom proportion increased from 63.64% to 79.38%. This observation indicates that different concentrations of carbon nanotubes were well incorporated and mixed with the CMC aerogels, resulting in the formation of the CNT-CMC aerogel.

**Figure 2:**
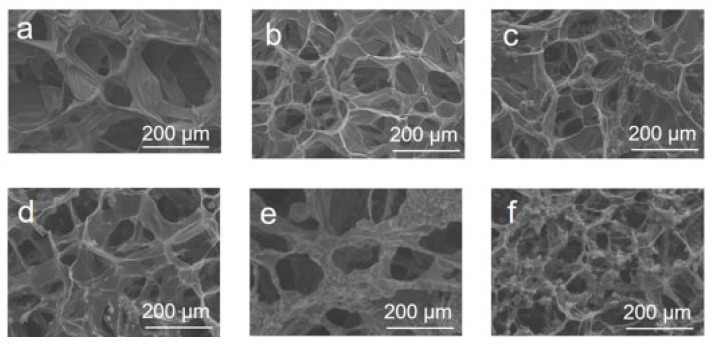
(**a**) SEM image of the surface of CMC aerogel. (**b**–**f**) SEM images of FT-CNT-CMC aerogel with carbon-nanotube concentrations of 5 mg/mL, 10 mg/mL, 15 mg/mL, 20 mg/mL, and 25 mg/mL, respectively.

Figure 2 depicts SEM images of the surface of CNT-CMC aerogels with carbon nanotubes concentrations ranging from 0 mg/mL to 25 mg/mL. The observed surface pore structure of the CNT-CMC aerogels in Figure 2a–f exhibits a well-maintained honeycomb shape, indicating the preservation of a three-dimensional porous structure. However, the interior of the 25 mg/mL CNT-CMC aerogel, as shown in Figure S3, displays a disordered and porous structure with poor maintenance. This is attributed to the collapse of the CMC aerogel skeleton to some extent due to the high carbon-nanotube content within the aerogel. These results suggest that CNT-CMC aerogels with carbon-nanotube concentrations up to 20 mg/mL maintain a good three-dimensional porous structure both on the surface and internally. 

**Figure 3:**
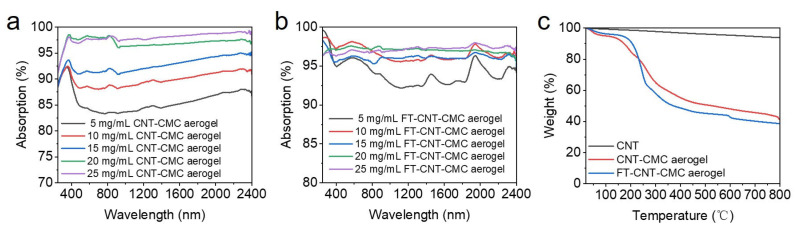
(**a**) UV-vis-NIR absorption spectra of CNT-CMC aerogel. (**b**) UV-vis-NIR absorption spectra of FT-CNT-CMC aerogel. (**c**) TGA of FT-CNT-CMC aerogel.

The increase in carbon nanotube content within the CNT-CMC aerogel is also associated with changes in the light reflectance, as measured by the UV-vis-NIR spectrometer (Figure S4a). The reflectance of the CNT-CMC aerogel within the 250–2400 nm range decreases from 16% at a carbon-nanotube concentration of 5 mg/mL to 3% at that of 25 mg/mL. Consequently, the absorption rate, calculated using the formula A = 1 − R, increases from 84% for the CNT-CMC aerogel with a carbon-nanotube concentration of 5 mg/mL to 97% for the one of 25 mg/mL (Figure 3a). These results clearly demonstrate the excellent light-absorbing properties of the CNT-CMC aerogel with carbon nanotubes. Notably, both the CNT-CMC aerogel with carbon-nanotube concentrations of 20 mg/mL and 25 mg/mL exhibit an absorption rate of 97% within the visible wavelength range of 300–800 nm, with no significant difference. Between the CNT-CMC aerogel with 20 mg/mL carbon-nanotube concentrations and that with 25 mg/mL, there is only a slight distinction of about 1% in the absorption rate within the infrared wavelength range, which indicates that the CNT-CMC aerogels with above 20 mg/mL carbon-nanotube concentrations possess similar light absorption properties and excellent light-absorbing capabilities. However, there is a collapse of the three-dimensional pore structure within the CNT-CMC aerogel with a carbon-nanotube concentration of 25 mg/mL (Figure S3f), which may lead to poor thermal insulation and increased heat loss in interfacial solar evaporation. Therefore, the CNT-CMC aerogel with a carbon-nanotube concentration of 20 mg/mL is considered to be the optimal sample.

Figure S4b illustrates the reflectance of FT-CNT-CMC aerogels with different carbon-nanotube concentrations, as measured by the UV-Vis-NIR spectrometer. The corresponding absorbance was calculated using the formula shown in Figure 3b. The reaction of Fe^3+^ in the CNT-CMC aerogel with tannic acid leads to the formation of black iron tannate, enhancing the light-absorbing properties of the aerogel. The increase in absorbance from 83% in the CNT-CMC aerogel with a carbon-nanotube concentration of 5 mg/mL to 92% in the FT-CNT-CMC aerogel was confirmed. However, the absorbance of both the CNT-CMC aerogel and the FT-CNT-CMC aerogel at a carbon-nanotube concentration of 20 mg/mL did not show a significant improvement, remaining at 96–98%. Although the light absorption capacity of the FT-CNT-CMC aerogel did not significantly increase after treating the CNT-CMC aerogel with tannic acid solution at a concentration of 20 mg/mL carbon nanotubes, this process plays a crucial role in achieving the double-layer evaporation structure, i.e., the upper hydrophilic layer and the bottom hydrophobic layer. The CNT-CMC aerogels treated with stearic acid solution exhibit hydrophobicity, while the reaction between tannic acid solution and Fe^3+^ in the hydrophobic CNT-CMC aerogel forms iron tannate, in which the introduction of a significant amount of hydrophilic groups (-OH) from tannic acid facilitates the formation of an interfacial evaporation structure with an upper hydrophilic layer. This superhydrophilic layer is conducive to the rapid dispersion of water droplets at the evaporation surface, which is helpful for improving the evaporation efficiency. Therefore, it is still necessary to react the CNT-CMC aerogel with tannic acid to obtain the FT-CNT-CMC aerogel.

The TGA diagrams of carbon nanotubes, CNT-CMC aerogels, and FT-CNT-CMC aerogels with a carbon-nanotube concentration of 20 mg/mL are presented in Figure 3c. It is evident that both CNT-CMC aerogels and FT-CNT-CMC aerogels exhibit two stages of mass loss. The mass loss observed in the FT-CNT-CMC aerogel between 27 and 100 °C can be attributed to the evaporation of absorbed water [30]. On the other hand, the mass loss occurring between 140 and 400 °C corresponds to the decomposition of CMC, which can be attributed to the loss of hydroxyl groups and potentially residual GDL [31]. Considering that the actual temperature during interfacial solar evaporation is much lower than 140 °C, this indicates that the FT-CNT-CMC aerogel demonstrates good thermal stability for interfacial solar evaporation.

**Figure 4:**
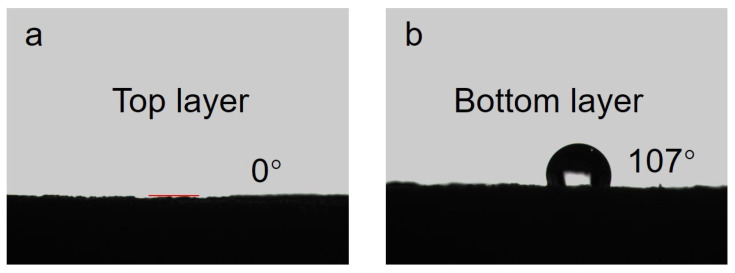
(**a**) Contact angle of the top layer of FT-CNT-CMC aerogel, and (**b**) contact angle of the bottom layer of FT-CNT-CMC aerogel.

Figure 4a displays the contact angle of the upper surface of the FT-CNT-CMC aerogel. The contact angle is 0°, indicating excellent wettability. This characteristic is advantageous for the even dispersion of water droplets dripping from above during the interface evaporation in the FT-CNT-CMC aerogel. In Figure 4b, the contact angle of the bottom surface of the FT-CNT-CMC aerogel is measured to be 107°. This high contact angle prevents the water from infiltrating from the upper layer. It works in conjunction with the three-dimensional porous structure of the aerogel, effectively insulating heat and minimizing heat loss [32].

**Figure 5:**
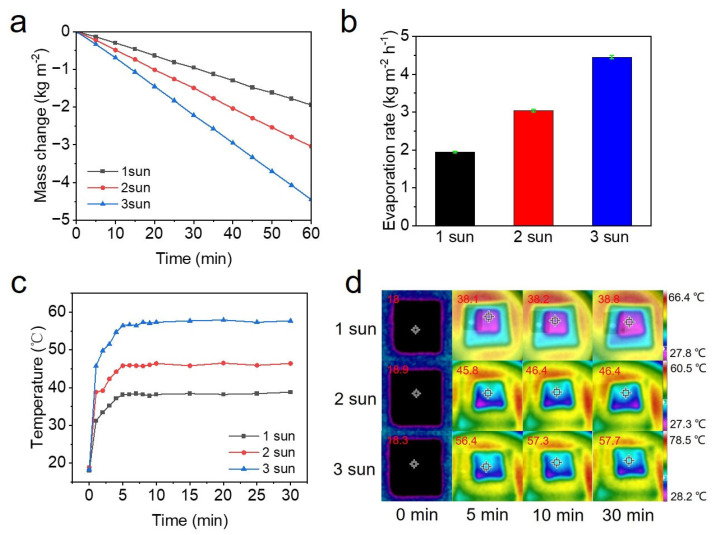
The FT–CNT–CMC aerogel with CNT concentration of 20 mg/mL was tested. (**a**) Variation of water mass with time under 1–3 light intensities. (**b**) Evaporation rate of water under 1–3 light intensities. (**c**) Surface temperature variation of FT–CNT–CMC aerogel under 1–3 light intensities. (**d**) Surface temperature of FT–CNT–CMC aerogel at different time intervals under 1–3 light intensities.

To evaluate the performance of the FT-CNT-CMC aerogel for solar interface photothermal evaporation, we conducted experiments to monitor the water quality changes over time under varying light intensities, as depicted in Figure 5a. From the evaporation rate data presented in Figure 5b, it is evident that the FT-CNT-CMC aerogel with a carbon-nanotube concentration of 20 mg/mL achieves a remarkable evaporation rate of 1.942 kg·m^−2^·h^−1^ under 1 sun light intensity, 3.04 kg·m^−2^·h^−1^ under 2 sun light intensities, and 4.449 kg·m^−2^·h^−1^ under 3 sun light intensities. These results highlight the efficient interfacial solar evaporation achieved with the incorporation of carbon nanotubes into the FT-CNT-CMC aerogel. As depicted in Figure 5c, the temperature of the FT-CNT-CMC aerogel rapidly increases over time under 1–3 sun light intensities, eventually stabilizing. In Figure 5d, it can be observed that the surface temperature of the FT-CNT-CMC aerogel raises from 18 °C to 38.1 °C within 5 min under 1 sunlight intensity. Under 2 sun light intensities, the surface temperature increases from 18.9 °C to 45.8 °C within 5 min, while under 3 sun light intensities, it rises from 18.3 °C to 56.4 °C after 5 min and then remains relatively stable at this temperature.

**Figure 6:**
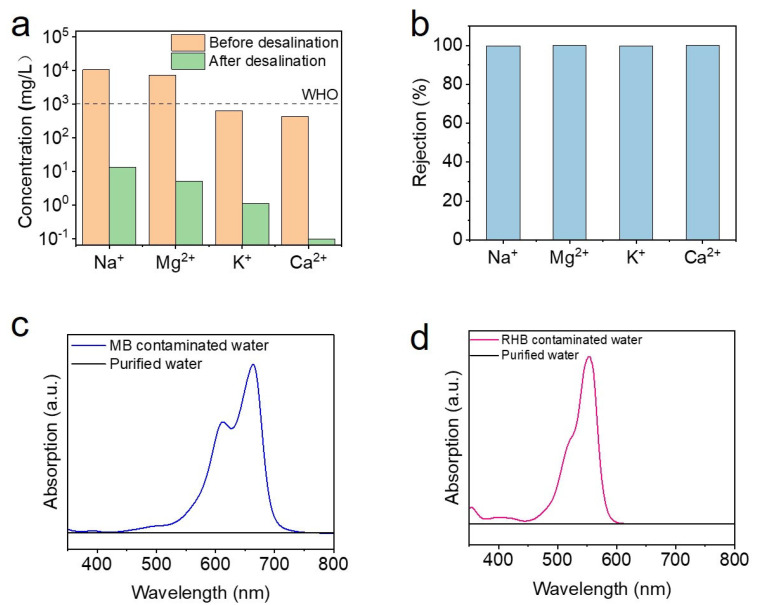
(**a**) Concentration of four salt ions in simulated seawater before and after purification by FT–CNT–CMC aerogel. (**b**) Retention rate of four salt ions after purification. (**c**) Absorption spectra of MB solution before and after purification by FT–CNT–CMC aerogel. (**d**) Absorption spectra of RHB solution before and after purification by FT–CNT–CMC aerogel.

To validate the practical application of FT-CNT-CMC aerogel in solar interface evaporation for seawater desalination, we conducted experiments using the FT-CNT-CMC aerogel to desalinate simulated seawater. The concentrations of Na^+^, Mg^2+^, K^+^, and Ca^2+^ ions were measured using ICP, as illustrated in Figure 6a. The results demonstrate a significant reduction in ion concentrations: Na^+^ ions decreased from 10,550 mg/L to 13.409 mg/L, Mg^2+^ ions decreased from 7190 mg/L to 5.12 mg/L, K^+^ ions decreased from 642.1 mg/L to 1.122 mg/L, and Ca^2+^ ions decreased from 432.2 mg/L to 0.097 mg/L. The concentration of these ions decreased by several orders of magnitude after desalination with FT-CNT-CMC aerogel, achieving a rejection rate exceeding 99% (Figure 6b). This confirms the effectiveness of FT-CNT-CMC aerogel for seawater desalination.

Furthermore, to assess the ability of the FT-CNT-CMC aerogel to purify organic dye wastewater, we performed solar energy interface evaporation on MB and RHB solutions. The absorption spectra of the solutions were measured using an ultraviolet-visible spectrophotometer, as shown in Figure 6c,d. After purification, the characteristic absorption peaks of MB solution at 660 nm and RHB solution at 552 nm disappeared [33,34], indicating the successful purification of the dye wastewater by the FT-CNT-CMC aerogel. The practical desalination effect on the simulated seawater and the purification of organic dye wastewater demonstrate the broad application prospects of the FT-CNT-CMC aerogel in solar energy interface evaporation applications.

## 4. Conclusions

Carbon nanotubes hold great promise as a light absorber for solar interfacial evaporation, owing to their exceptional thermal conductivity and light absorption properties of the entire solar spectrum. Moreover, CNT is cost-effective, scalable, and adaptable as a photothermal material. To enhance the performance of solar energy interfacial evaporation, a double-layer aerogel structure was designed in this study. The rich natural carboxymethyl cellulose was used as the basic skeleton to achieve sustainability and biodegradability. The FT-CNT-CMC aerogel was prepared with carbon nanotubes as the photothermal material. The developed FT-CNT-CMC aerogel shows excellent optical absorption performance, with an absorption rate of more than 96% in the range of 250–2400 nm. In addition, under 1 solar irradiation, the evaporation rate reached an impressive 1.942 kg·m^−2^·h^−1^, and the photothermal conversion efficiency reached 92.8%. It is worth noting that the composite aerogel has the advantages of being low cost, renewable, and a simple preparation process. These characteristics make it have practical application prospects in solar desalination.

## Data Availability

The data that support the findings of this study are available from the corresponding author upon reasonable request.

## Supplementary Materials

The following supporting information can be downloaded at: https://www.mdpi.com/article/10.3390/ma16175815/s1, Figure S1: Experimental setup diagram of solar steam generation; Figure S2: (a–e) SEM images of carbon nanotube/carboxymethyl cellulose aerogels with carbon nanotube concentrations of 5 mg/ml, 10 mg/ml, 15 mg/ml, 20 mg/ml, and 25 mg/ml, respectively. (f) Carbon atomic ratio in carbon nanotube/carboxymethyl cellulose aerogels with different carbon nanotube concentrations as scanned by EDS; Figure S3: (a) SEM image of the internal structure of CMC aerogel. (b–f) SEM images of the internal structure of CNT-CMC aerogels with carbon nanotube concentrations of 5 mg/ml, 10 mg/ml, 15 mg/ml, 20 mg/ml, and 25 mg/ml, respectively; Figure S4: (a) Reflectance of CNT-CMC aerogels with carbon nanotube concentrations of 5 mg/ml, 10 mg/ml, 15 mg/ml, 20 mg/ml, and 25 mg/ml. (b) Reflectance of FT-CNT- CMC aerogels with carbon nanotube concentrations of 5 mg/ml, 10 mg/ml, 15 mg/ml, 20 mg/ml, and 25 mg/ml; The evaporation conversion efficiency; Table S1: Comparison of solar evaporation performance of FT-CNT-CMC aerogel with other recent reported solar evaporators based on wood or cellulose aerogel; Figure S5: Indoor solar evaporation and water collection setup. (a) Desalination of seawater. (b) Purification of MB solution. (c) Purification of RHB solution; Salt resistance cycling test of the FT-CNT-CMC aerogel; Figure S6: Evaporation rates of the FT-CNT-CMC aerogel-based device under illumination of 1 kW/m2 for 6 salt resistant cycles; Figure S7: (a,b) SEM of FT-CNT-CMC aerogel after 5 salt resistant cycles; (c,d) SEM of FT-CNT-CMC aerogel after cleaning after 5 salt resistant cycles; Table S2: List of nomenclatures; Video S1: Contact angle of the FT-CNT-CMC aerogel.
