# Supplementary material for: Efficient Solar Desalination of Seawater Using a Novel Carbon Nanotube-Based Composite Aerogel
Source: Materials (Basel). 2023 Aug 24;16(17):5815. doi: 10.3390/ma16175815 (PMC10488654; doi:10.3390/ma16175815)
Supplement: Supplementary file 1 [file materials-16-05815-s001.zip › materials-2527544-SI.pdf]

Efficient Solar Desalination of Seawater using a Novel Carbon Nanotube-Based Composite Aerogel

Shuai Liu [1], Shun Wang [1], Shunxu Shuai [1], Yuyan Weng [1] and Fengang Zheng [1,2,*]

[1] Jiangsu Key Laboratory of Thin Films, School of Physical Science and Technology, Soochow University, Suzhou 215006, China; 20204208039@stu.suda.edu.cn (S.L.); wengyuyan@suda.edu.cn (Y.W.)
[2] SJTU-Pinghu Institute of Intelligent Optoelectronics, Jiaxing 314200, China
* Correspondce: zhfg@suda.edu.cn

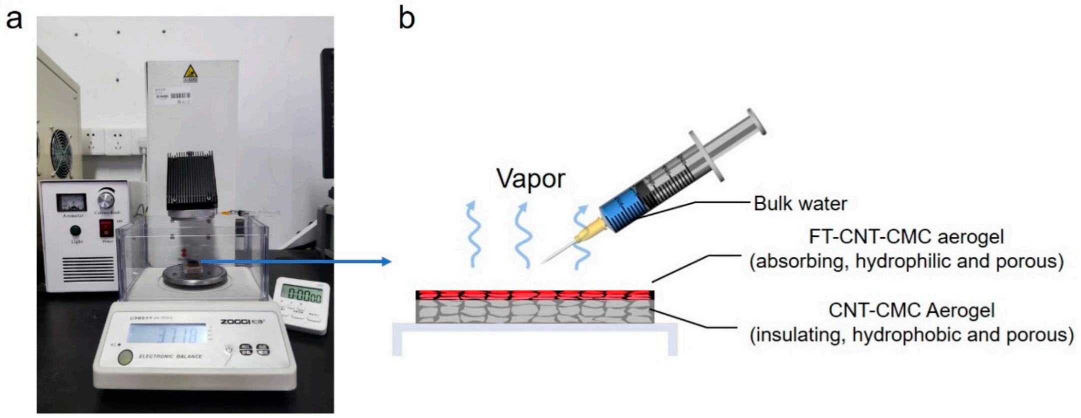

Figure S1. Experimental setup diagram of solar steam generation.

Figure S1 is an experimental setup diagram of solar steam generation, which adjusts the light intensity by adjusting the current of the xenon lamp to achieve different light intensities of 1 sun, 2 sun, and 3 sun.

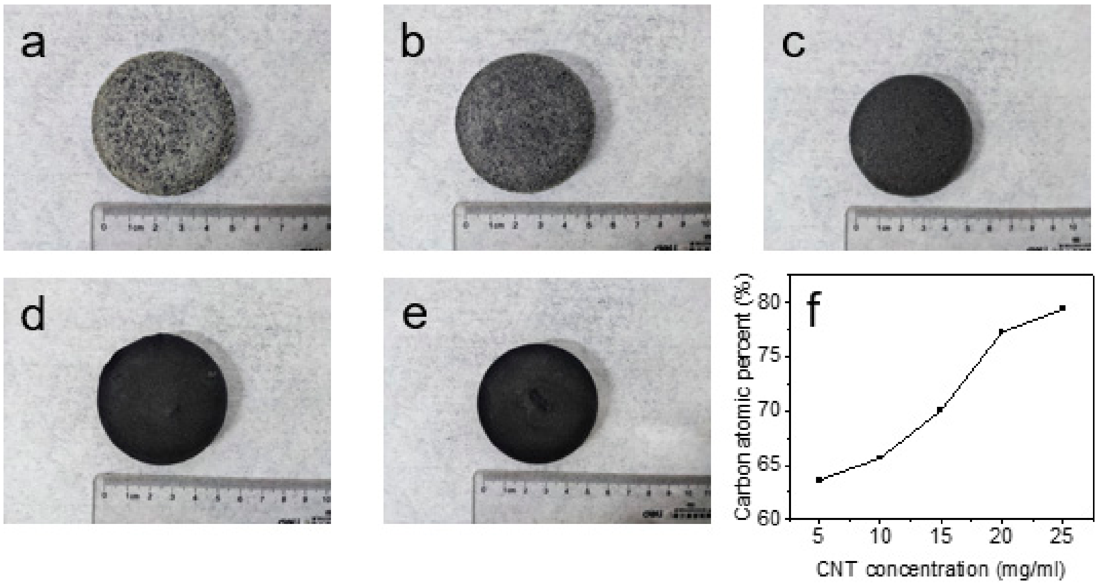

Figure S2. (a-e) SEM images of carbon nanotube/carboxymethyl cellulose aerogels with carbon nanotube concentrations of 5 mg/ml, 10 mg/ml, 15 mg/ml, 20 mg/ml, and 25 mg/ml, respectively. (f) Carbon atomic ratio in carbon nanotube/carboxymethyl cellulose aerogels with different carbon nanotube concentrations as scanned by EDS.

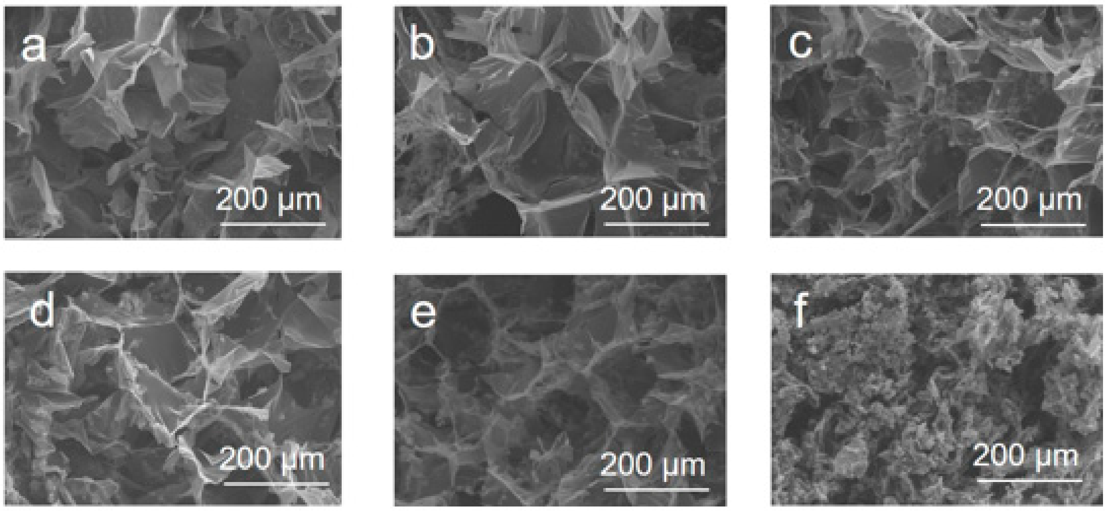

Figure S3. (a) SEM image of the internal structure of CMC aerogel. (b-f) SEM images of the internal structure of CNT-CMC aerogels with carbon nanotube concentrations of 5 mg/ml, 10 mg/ml, 15 mg/ml, 20 mg/ml, and 25 mg/ml, respectively.

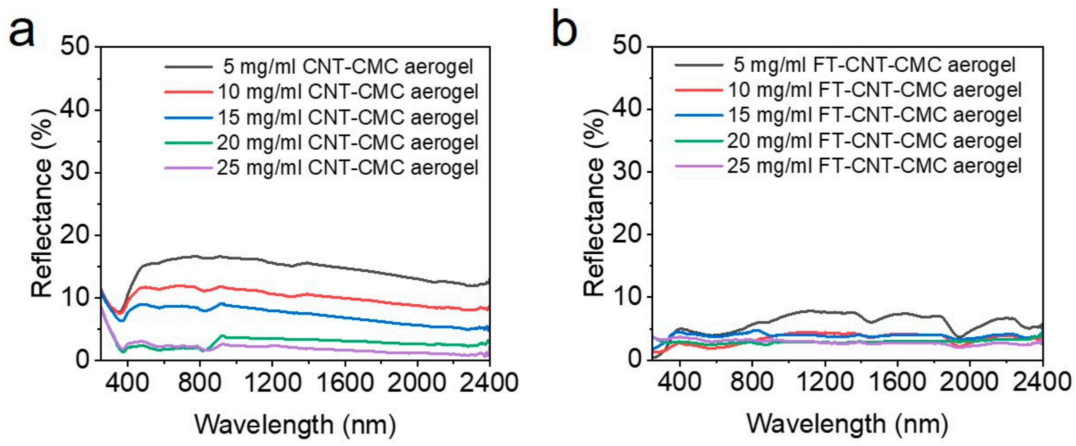

Figure S4. (a) Reflectance of CNT-CMC aerogels with carbon nanotube concentrations of 5 mg/ml, 10 mg/ml, 15 mg/ml, 20 mg/ml, and 25 mg/ml. (b) Reflectance of FT-CNT-CMC aerogels with carbon nanotube concentrations of 5 mg/ml, 10 mg/ml, 15 mg/ml, 20 mg/ml, and 25 mg/ml.

The evaporation conversion efficiency

The evaporation conversion efficiency, η, is calculated as follows:

$$\eta = \frac{P_e}{P_s} \quad \text{(S1.1)}$$

where P_s is the intensity of solar irradiation (1KW/m^2), and P_e is the power used for water evaporation. The power for water evaporation is defined as:

$$P_e = E_1 m_1 \quad \text{(S1.2)}$$

Wherein m_1 is the solar evaporation rate of FT-CNT-CMC aerogel and E_1 is the equivalent vaporization enthalpy of water in FT-CNT-CMC aerogel.

To calculate the equivalent vaporization enthalpy of water in FT-CNT-CMC aerogel (E_1), we hypothesized that under the same ambient temperature and pressure, the evaporation rate and vaporization enthalpy of pure water and FT-CNT-CMC aerogel satisfy the following equation:

$$E_0 m_0 = E_1 m_1 \quad \text{(S1.3)}$$

where m_1, E_0 and m_0 are the evaporation rate in FT-CNT-CMC aerogel, the vaporization enthalpy of pure water, and the evaporation rate of pure water, respectively. Thus, the

equivalent vaporization enthalpy of water in FT-CNT-CMC aerogel can be estimated as:

$$E_1 = \frac{m_0}{m_1} E_0 \tag{S1.4}$$

Using the reported vaporization enthalpy of pure water (∽2256 J/g), the evaporation conversion efficiency of FT-CNT-CMC aerogel can be calculated as 92.8%.

Table S1. Comparison of solar evaporation performance of FT-CNT-CMC aerogel with other recent reported solar evaporators based on wood or cellulose aerogel.

Ref.	Substrate	Photothermal materials	Evaporation rate ($kg\cdot m^{-2}\cdot h^{-1}$)	Efficiency (%)	Year
This work	Cellulose aerogel	CNT	1.942	92.8	2023
1	Wood	Fe_3O_4	1.39	90.6	2021
2	Wood	Surface carbonization	1.46	75	2019
3	Cellulose aerogel	Polydopamine	1.36	86	2021
4	Cellulose/alginate/ CB hydrogel	Carbon black	1.33	90.6	2022
5	Dialdehyde MCC membrane	CNT	1.58	90.86	2022
6	Cellulose hydrogel	Carbon black	1.582	91.40	2020
7	NFC aerogel	PEDOT:PSS	1.61	81	2020
8	Cellulose-based aerogel	PPy	1.66	94.62	2022
9	Cellulose	CNT	1.9	89.16	2021
10	Cellulose artificial wood	CNT	1.76	85	2022

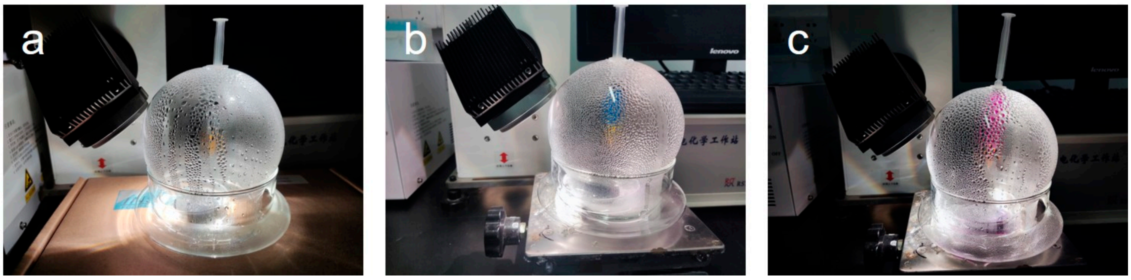

Figure S5. Indoor solar evaporation and water collection setup. (a) Desalination of seawater. (b) Purification of MB solution. (c) Purification of RHB solution.

Salt resistance cycling test of the FT-CNT-CMC aerogel

We conducted tests on the salt resistance of FT-CNT-CMC aerogel, as depicted in Figure S6. After four cycles, there was a noticeable decrease in the evaporation rate during the fifth cycle. This reduction was attributed to the accumulation of salt ions on the surface of the FT-CNT-CMC aerogel after multiple tests. Subsequently, rinsing the FT-CNT-CMC aerogel surface with deionized water helped to remove some of the salt ions, as depicted in Figure S7. Following the rinsing process, the evaporation performance of the FT-CNT-CMC aerogel showed a certain degree of recovery during the sixth cycle test.

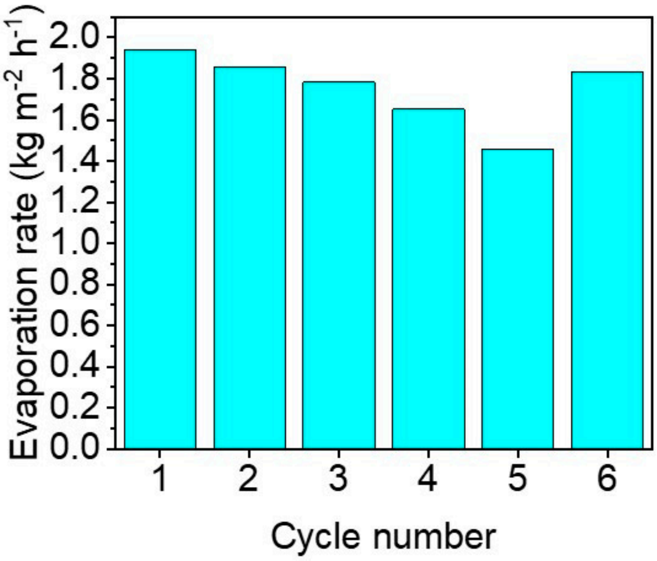

Figure S6. Evaporation rates of the FT-CNT-CMC aerogel-based device under illumination of 1kW/m^2 for 6 salt resistant cycles.

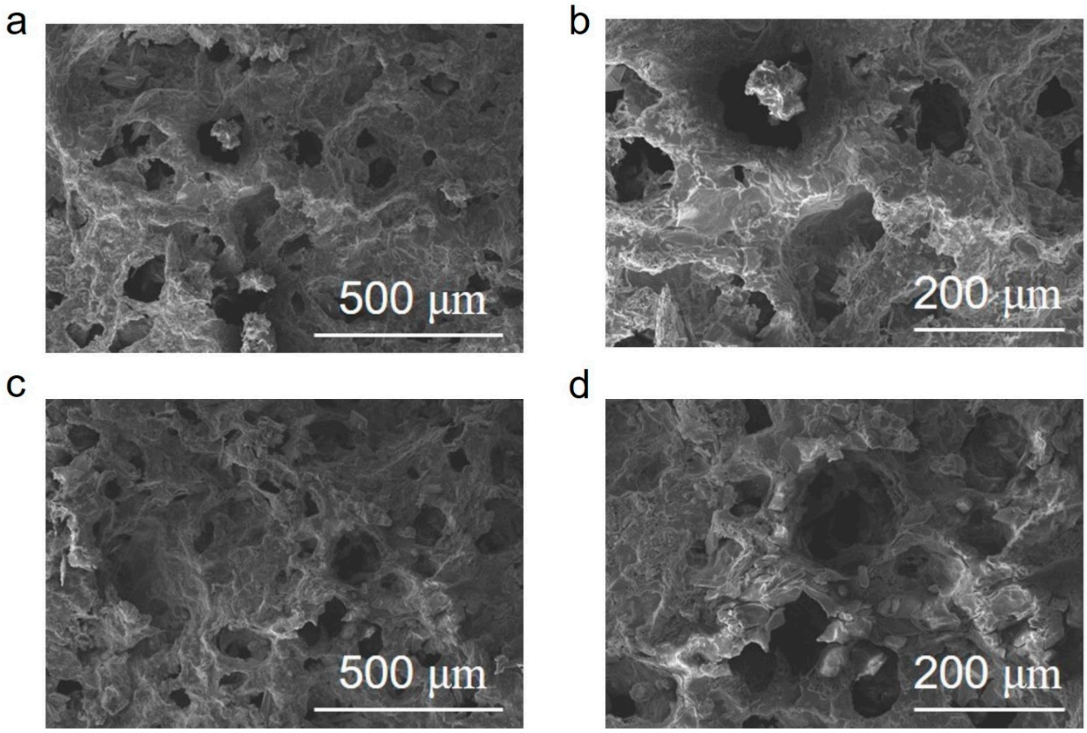

Figure S7. (a, b) SEM of FT-CNT-CMC aerogel after 5 salt resistant cycles; (c, d) SEM of FT-CNT-CMC aerogel after cleaning after 5 salt resistant cycles.

Table S2. List of nomenclatures.

Full name	Abbreviation
solar steam generation	SSG
carbon nanotube	CNT
carboxymethyl cellulose	CMC
ferric tannate/carbon nanotube/carboxymethyl cellulose composite aerogel	FT-CNT-CMC aerogel
D-(+)-gluconic acid-lactone	GDL
methylene blue	MB
rhodamine B	RHB
carbon nanotube/carboxymethyl cellulose aerogel	CNT-CMC aerogel
scanning electron microscopy	SEM
energy-dispersive X-ray spectroscopy	EDS
thermogravimetric analyzer	TGA
ultraviolet-visible-near-infrared	UV-Vis-NIR
Contact angles	WCA
inductively coupled plasma emission spectrometry	ICP

Video S1
Contact angle of the FT-CNT-CMC aerogel.

References

1. Gan, W.; Wang, Y.; Xiao, S.; Gao, R.; Shang, Y.; Xie, Y.; Liu, J.; Li, J. Magnetically driven 3D cellulose film for improved energy efficiency in solar evaporation. *ACS Applied Materials & Interfaces*, **2021**, 13(6): 7756.
2. Kuang, Y.; Chen, C.; He, S.; Hitz, E. M.; Wang, Y.; Gan, W.; Mi, R.; Hu, L.; A high-performance self-regenerating solar evaporator for continuous water desalination. *Advanced materials*, **2019**, 31(23): 1900498.
3. Zou, Y.; Zhao, J.; Zhu, J.; Guo, X.; Chen, P.; Duan, G.; Liu, X.; Li, Y. A mussel-inspired polydopamine-filled cellulose aerogel for solar-enabled water remediation. *ACS Applied Materials & Interfaces*, **2021**, 13(6): 7617.
4. Yuan, J.; Lei, X.; Yi, C.; Jiang, H.; Liu, F.; Cheng, G. J. 3D-printed hierarchical porous cellulose/alginate/carbon black hydrogel for high-efficiency solar steam generation. *Chemical Engineering Journal,* **2022**, 430: 132765.
5. Zhu, R.; Wang, D.; Liu, Y.; Liu, M.; Fu, S. Bifunctional superwetting carbon nanotubes/cellulose composite membrane for solar desalination and oily seawater purification. *Chemical Engineering Journal*, **2022**, 433: 133510.
6. Hu, N.; Xu, Y.; Liu, Z.; Liu, M.; Shao, X.; Wang, J.; Double-layer cellulose hydrogel solar steam generation for high-efficiency desalination. *Carbohydrate polymers,* **2020**, 243: 116480.
7. Han, S.; Ruoko, T. P.; Gladisch, J.; Erlandsson, J.; Wagberg, L.; Crispin, X.; Fabiano, S. Cellulose-conducting polymer aerogels for efficient solar steam generation. *Advanced Sustainable Systems*, **2020**, 4(7): 2000004.
8. Zhu, R.; Wang, D.; Xie, J.; Liu, Y.; Liu, M.; Fu, S. Salt-resistant Schiff base cross-linked superelastic photothermal cellulose aerogels for long-term seawater desalination, *Chemical Engineering Journal*, **2022**, 427: 131618.
9. K. Liu, W. Zhang, H. Cheng, L. Luo, B. Wang, Z. Mao, X. Sui, X. Feng, A nature-inspired monolithic integrated cellulose aerogel-based evaporator for efficient solar desalination. *ACS Applied Materials & Interfaces*, **2021**, 13(8): 10612.
10. Zhang, X.; Zhang, T.; An, X.; Li, M.; Pei, D.; Zhang, J.; Li, C. Guiding cellular channels of artificial nanohybrid woods for anisotropic properties and solar-thermal evaporation. *Chemical Engineering Journal*, **2022**, 428: 132060.
